# Early feasibility testing of a web-based mind-body resiliency program for adults with neurofibromatosis: The NF-Web study

**DOI:** 10.1016/j.pecinn.2022.100076

**Published:** 2022-08-20

**Authors:** Ethan G. Lester, Nathan S. Fishbein, Annabel Peterson, Ana-Maria Vranceanu

**Affiliations:** a The Center for Health Outcomes and Interdisciplinary Research (CHOIR), Massachusetts General Hospital, Boston, MA, United States of America; b Harvard Medical School, Boston, MA, United States of America

**Keywords:** Psycho-oncology, Neurofibromatosis, Quality of life, Stress management, Mind-body, Web-based, Resiliency

## Abstract

**Objectives:**

We aimed to test early feasibility, acceptability, and signals of improvement after the 8-week individually delivered asynchronous, web-based mind-body program (NF-Web) modeled after the synchronous group-based live video program (Relaxation Response Resiliency Program for NF; 3RP-NF).

**Methods:**

Two cohorts (cohort 1 *n* = 14, cohort 2 *n* = 14) completed baseline and posttest (feasibility markers, *t-*tests).

**Results:**

Enrolled participants (*N* = 28; 80% of those eligible) completed baseline (N = 28; 100% of sample) and posttests (*N* = 25; 89.3%). Video lesson (58.0%) and homework (70.9%) completion were fair to good. Satisfaction (*M* = 8.85/10; SD = 2.35), credibility (*M* = 7.07/10; SD = 1.44), and expectancy (*M* = 6.68/10; *SD* = 2.10) were good to excellent. Participation was associated with statistically significant pre-to-post positive changes in quality of life (QoL; Physical, Psychological, Social, and Environmental,; *p* < 0.05) and emotional distress (depression, anxiety, and stress; *p* < 0.05). Pain intensity and interference did not improve significantly (*p* > 0.05) after participation.

**Conclusions:**

NF-Web demonstrates initial feasibility, acceptability, and signals of improvement. Results support future trials to ascertain efficacy.

**Innovation:**

Web-based programs may be valuable for individuals with rare illness who prefer to learn skills on their own timeline, have barriers to live video participation, and who also have apprehensions about interacting with others during treatment.

## Highlights

• A website intervention for adults with neurofibromatosis (N = 28) was feasible.
• Participation was associated with pre/post QoL and emotional distress improvements.
• Psychosocial web-programs for rare illnesses may be useful and warrant attention.

## 1 Introduction

Neurofibromatoses (NF; including NF1, NF2, and schwannomatosis) are a group of rare neurogenetic disorders (NF1 occurs in approximately 1:2700 live births; NF2 and schwannomatosis occurs in approximately 1:25,000–33,000 life births, respectively). The three NF types are biomedically district disorders, with individuals experiencing varying degrees of physical symptoms burden, including disabling and disfiguring cutaneous tumors, gait issues, and learning disabilities in NF1, hearing loss and tinnitus in NF2, and chronic pain in schwannomatosis [ [1], [2], [3], [4], [5]]. Unfortunately, there are no curative interventions for NF, with treatment options primarily being surgical, palliative [6], and more recently pharmacological (e.g., use of MEK inhibitors in NF1). Although biologically distinct, people with NF are unified by a similar psychosocial profile marked by chronic NF-specific stressors, including frequent medical appointments and surgeries, ongoing fears of tumor malignancies and other related illnesses, and concerns about prognoses and passing the illness to their offspring [7]. Given these significant challenges, individuals with NF may experience clinically significant levels of emotional distress (e.g., depression, anxiety, stress) [6,8], lower self-esteem and greater social difficulties [9], and reduced quality of life (QoL) compared to the general population [10].

Very few NF-specific psychosocial treatment options exist and there are limited mental health providers trained to deliver these treatments [[11], [12]]. To address this need, we developed the adult (18+) live video mind-body resiliency programs that aimed to improve QoL (physical, psychological, social, and environmental), emotional distress (anxiety, depression, stress), as well as resiliency factors (e.g., mindfulness, gratitude, optimism, coping) called the Relaxation Response Resiliency Program for NF (3RP-NF) [11]. The program was designed to be delivered virtually through live video conferencing software (e.g., Zoom) over the course of 8 weekly 90-min sessions, where participants meet with other individuals with NF and learn mind-body and resiliency skills. Participants are asked to practice these skills during the week and keep track with homework logs reviewed at the beginning of session. This trial included individuals across the globe and exhibited high levels of feasibility and acceptability. Participation in the intervention group was associated with improvements in QoL domains, emotional distress, and pain compared to population-matched health education attention placebo control [11].

Despite this program's success, we quickly learned about important barriers that prevented individuals from participating in live video groups. In fact, roughly 1/3 participants expressing interest in the live video program trial program could not participate due to specific barriers [12] including: (1) time-zone differences, (2) scheduling conflicts due to occupational/familial obligation, and (3) NF-specific physical appearance concerns which lead to on-screen participation avoidance [12,13]. Given these barriers and the need for larger-scale implementation, innovations in treatment approaches are needed. Website-based (“web-based”) platforms for treatment are a promising approach as they have enhanced accessibility and are often implemented to address systemic barriers to care (e.g., cost, convenience, stigma) [[14], [15]]. Web-based interventions with similar treatment approaches (e.g., mindfulness-based interventions) have shown benefits for adult populations experiencing health related issues [16], and, therefore, exploration of a web-based treatment approach in NF is warranted [12,13,17].

To enhance accessibility and sustainability of our program, we developed an individually delivered asynchronous web-based platform, “*NF-Web*,” adapted directly from the live video, group-based 3RP-NF resiliency program [11,12]. Here, we describe the early feasibility testing of NF-Web, including acceptability and signals of improvements (within-group) for QoL, emotional distress, and pain outcomes. We hypothesized that NF-Web would demonstrate early feasibility by meeting a-priori feasibility marks and that NF-Web would be positively associated with within-group changes in secondary outcomes (QoL, emotional distress, and pain) from baseline to posttest.

## 2 Methods

### 2.1 Participants

**Fig. 1:**
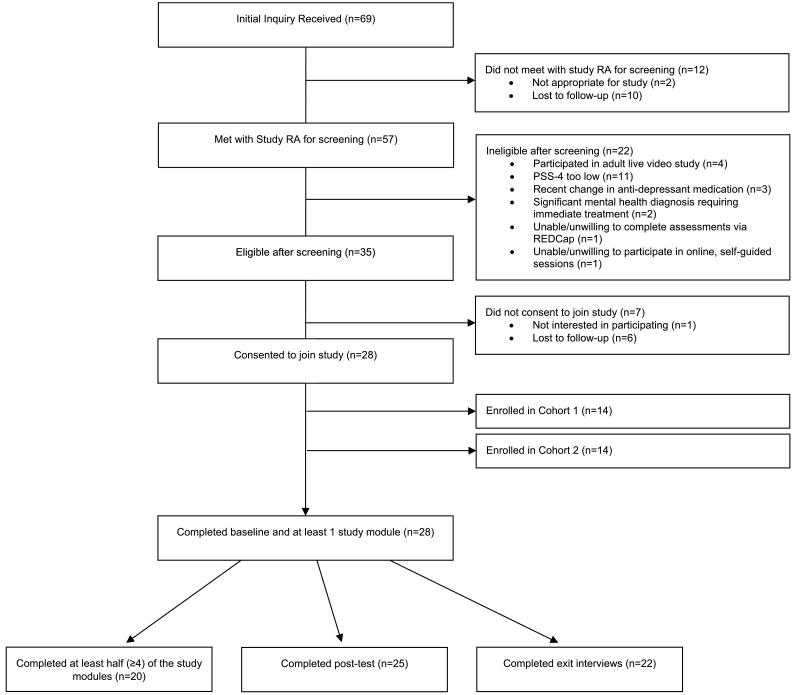
CONSORT Diagram for the NF-Web Study

We enrolled two separate participant cohorts across the study period (10/1/2020–5/13/2021). This approach helped us assess and optimize administrative support for NF-Web (e.g., website support, exit interview scheduling) and allowed for simulation of a cohort-based delivery format. For this study, we recruited participants from two sources 1) adults who could not participate in the live video 3RP-NF program, and 2) from our funding foundations' (NF Northeast, NF Midwest, Texas Neurofibromatosis Foundation, and the Children's Tumor Foundation) through emailed newsletters, foundation announcements and websites. We successfully recruited 28 participants across two cohorts (14 each) to take part in the eight-week NF-Web trial. Inclusion/exclusion criteria for this trial are presented in Table 1 (See Fig. 1).

**Table 1:** Inclusion/Exclusion Criteria for the NF-Web Trial.

Inclusion/Exclusion Criteria for NF-Web

• Score of 6 or higher on the Perceived Stress Scale v.4 (PSS-4)
• Adults aged 18 years or older
• Diagnosis of neurofibromatosis (NF; NF1, NF2, or schwannomatosis)
• English-speaking and self-reported literacy at 6th grade reading level or above
• Self-reported difficulties coping with stress and NF symptoms
• No change in antidepressant medication in the past 3 months
• No Cognitive Behavioral Therapy or relaxation therapy in the past 3 months
• No medical comorbidity expected to worsen in the next 12 months
• Able to complete questionnaires online and participate in live video interventions
• Did not participate in previous adult live video program (3RP-NF).

### 2.2 NF-Web intervention

The NF-Web program is an eight-week, asynchronous web-based mind-body program adapted directly from the live video 3RP-NF mind-body group intervention for adults with NF [11]. NF-Web content is the same content as the live video 3RP-NF program, with website adaptation (e.g., format, media) informed by 3RP-NF participant feedback [12]. NF-Web includes 8 treatment modules (modules synonymous with 3RP-NF sessions) completed over 8 weeks (1 module/week; introduction and training module completed after baseline assessment prior to starting the program). The NF-Web intervention differs from the 3RP-NF live video program in two specific ways: 1) no live program delivery from a trained therapist, and 2) no live interaction with other people who have NF.

Participants completed treatment modules on a weekly basis at their own pace and convenience. Participants were given a personalized account and unique login and study administrators “unlocked” (i.e., released content on the website) new modules each week. Modules included pre-recorded video lessons taught by a licensed clinical psychologist (EGL), audio recordings for practice, and written transcripts (for individuals with hearing differences and/or deafness). The psychologist taught resiliency skills from the 3RP-NF in each session, including eliciting the relaxation response (e.g., mindfulness, diaphragmatic breathing, and body scans), stress awareness (e.g., identifying stress warning signals, thought distortions, and negative automatic thoughts), coping strategies (e.g., adaptive thinking, problem solving, and acceptance), and healthy lifestyle behaviors (e.g., healthy eating, sleep, and physical activity). Modules typically contained 6–10 short video lessons (5–10 min) when completing in one sitting would take approximately 30–45 min to complete. With videos embedded on NF-Web from another video platform site (i.e., YouTube), we were not able to assess how much time was spent on each video, only when a video lesson was opened on NF-Web and completed. For comprehension and accountability, we asked participants to complete a 5-question quiz on the materials each week on the website. Participants also submitted weekly homework assignments through the website portal each week, which included a relaxation practice and coping log (including time practiced, date, activity), and a daily gratitude and appreciation list. For a weekly module to be considered completed, participants would need to watch more than half the lesson videos and complete the quiz at the end of the module.

The NF-Web platform was designed so that participants could login to the website anytime during the week for their assigned module. From the home screen, participants navigated to specific website tabs at the top of the website browser which directed them to *Sessions, Discussion* (discussion board), *Frequently Asked Questions, Resources, Terms and Conditions,* an *About Us* page, *Contact,* and their personal *Profile* page. Participants primarily used the *Sessions* tab and completed their specific assignments for that week – including video and audio lessons, quizzes (example quiz questions, “Which of the following is a NF-Web tip for improving sleep?” and “What does the acronym NAT stand for?”), and session summaries. Table 2 displays the weekly NF-Web modules and lessons.

**Table 2:** NF-Web Modules and lessons displayed by week (8-week program).

NF-Web Modules with Specific Video Lessons (top to bottom/two columns)	
Introduction	
NF-Web: The Basics	NF-Web Mobile Friendly Tour
NF-Web Tutorial (Video)	NF-Web Session Summary
NF-Web Resource Page	Brief Introduction Quiz
**Week 1: Symptom Management, Stress Management, and Resiliency Training**	
The Stress Response	Gratitude and Appreciation
The Relaxation Response	Home Practice
The Mind Body Connection	Virtual Classroom
Single Point Focus Meditation	Session Summary Sheet
The Energy Battery	Session 1 Quiz⁎
Setting Smart Goals	
**Week 2: The Relaxation Response**	
Review	Identifying Emotions & Physical Sensations
The Body Scan	Home Practice
The MINIs	Virtual Classroom
Recuperative Sleep	Session Summary Sheet
Sleep Tips	Session 2 Quiz⁎
The RR and Emotions	
**Week 3: Stress and Symptom Awareness for Patients with Neurofibromatosis**	
Review	Social Support Diagram
Mindfulness	Mindful Body Awareness
Mindful Awareness Meditations	Home Practice
Mindful Eating	Virtual Classroom
Stress Response Components	Session Summary Sheet
Stress Warning Signals	Session 3 Quiz⁎
Social Support	
**Week 4: Mending Mind and Body of Patients with Neurofibromatosis**	
Review	Introduction to Adaptive Thinking
Movement to Elicit the RR	Home Practice
Negative Automatic Thoughts	Virtual Classroom
Thought Distortions	Session Summary Sheet
Emotions and Underlying Beliefs	Session 4 Quiz⁎
**Week 5: Creating an Adaptive Perspective**	
Review	Joyful Place Imagery
Guided Imagery	Pleasant Activities
Adaptive Thinking (Part 2)	Home Practice
Creating Adaptive Perspectives	Virtual Classroom
Healthy Eating	Session Summary Sheet
Stop, Breathe, Reflect, Choose	Session 5 Quiz⁎
**Week 6: Promoting Positivity**	
Review	Physical Activity
Loving Kindness Meditation	Home Practice
Telling Our Stories with Optimism	Virtual Classroom
Optimism vs Pessimism	Session Summary Sheet
Relaxation Signals	Session 6 Quiz⁎
**Week 7: Healing States of Mind**	
Review	E.M.P.A.T.H.Y Exercise
Contemplation	Letter to Self
Problem Solving and Acceptance	Virtual Classroom
PS-A Decision Tree	Home Practice
Finding Acceptance	Virtual Classroom
Empathy and Compassion	Session Summary Sheet
Mindful Awareness of Another	Session 7 Quiz⁎
**Week 8: Humor, Empathy, and Staying Resilient**	
Review	Reflecting on Resiliency Skills
Guided Imagery (Idealized Self)	Making a SMART Resiliency Plan
Humor	Wrap Up
Laughter	Virtual Classroom
More Humor Strategies	Session Summary Sheet
Staying Resilient	Session 8 Quiz⁎
Resiliency Skill Review	

⁎ In addition to weekly quizzes, all lessons after the introduction concluded with a direct website link to a homework log submission portal and online discussion board with prompting questions.

In addition to weekly modules, participants had the option to participate in an anonymous discussion board which allowed them to connect with other participants during the program. Discussion board participation was monitored weekly by study staff but was not used for these specific study outcomes. Participants also had access to a “Frequently Asked Questions” (FAQs) webpage integrated into the website. FAQs included questions about NF-Web, study participation, and psychosocial resources (program manual, skills worksheets, NF foundations and mental health resources). Participants were also able to ask questions directly to the study staff on a contact page.

### 2.3 Procedures

The Massachusetts General Hospital Institutional Review Board (IRB) approved all study procedures. Prior to beginning the program, participants were emailed the baseline assessments through REDCap (a secure electronic research data collection software) [18]. After participants completed baseline measures, we asked participants to complete the introduction module to help train how to use the website. After completing the introduction module, we asked participants to begin completing weekly NF-Web modules for 8 weeks as we assigned them.

Participants who did not complete the first NF-Web module within 4 days of the first week were called by a study coordinator to assist with completion, as well as any help with any relevant technical issues. Throughout the study, study staff called, texted, and/or emailed participants (with their consent) who had missed two or more sessions in a row. Most of these contact attempts were not returned (voicemails and messages left). Participants who did respond often mentioned “forgetting” the session assignment, as well as limited time and motivation. Study staff routinely delivered reinforcement and encouragement via email weekly when homework completion and website interaction occurred. We administered weekly assessments during the eight weeks (abbreviated measures – not analyzed as part of the current study). We recorded survey completion, weekly modules/lessons completion, discussion board participation, and homework completion in an external study log and verified against weekly website data.

After completing the program, participants completed posttest assessments through the same REDCap system [18]. At the end of the study, we also asked participants to complete an optional semi-structured exit interview by telephone where we assessed satisfaction/dissatisfaction with the intervention and suggestions for improvements (not analyzed as part of the current study).

### 2.4 Measures

#### 2.4.1 Primary outcomes

Primary outcomes included feasibility (including enrollment, measurement, and treatment adherence) and acceptability (including satisfaction, credibility, expectancy). Benchmarks and qualifiers were based on our previous clinical trials (Table 3) [11,19,20]. Notably, we collected these data passively (e.g., completion of session content, weekly homework submission) and actively (e.g., survey completion).

**Table 3:** Feasibility and Acceptability Markers for the NF-Web Study.

Feasibility and Acceptability Markers	Definition	Benchmarks and Qualifiers	NF-Web Outcome *(N; %)* *(M; SD)*
**Enrollment**	The total number of interested participants eligible to participate who enrolled divided by the total number who were interested and eligible but did not enroll	**80+% - Excellent** **70–79% - Good** **50–70% - Fair** **<50% - Poor**	*N=* 28; 80.0%
**Measurement**	The total number of participants who completed posttest measurement divided by the total number who completed baseline measurement	**100% - Excellent** **75–99% - Good** **60–74% - Fair** **<60% - Poor**	*N=* 25, 89.3%
**Treatment Adherence**	The total number of participants who completed at least half of the modules divided by the total number of participants enrolled in NF-Web The total number of video lessons completed by all participants divided by the total number video lessons assigned in the NF-Web program The total number of homework assignments completed divided by the total number of homework assignments in the NF-Web program	**80+% Excellent** **70–79% Good** **50–70% Fair** **<50% Poor**	*N=* 20; 71% 1218/2100, 58.0% 159/224, 70.9%
**Satisfaction**	The mean satisfaction score for the sample as measured by the Client Satisfaction Questionnaire	***M* = 8+− Excellent** ***M* = 6–7.99 - Good** ***M ≤6* - Poor**	*M=* 8.85; *SD=* 2.35
**Credibility**	The mean credibility score for the sample as measured by the Credibility and Expectancy Questionnaire	***M* = 8+− Excellent** ***M* = 6–7.99 - Good** ***M ≤6* - Poor**	*M=* 7.07; *SD=* 1.44
**Expectancy**	The mean expectancy score for the sample as measured by the Credibility and Expectancy Questionnaire	***M* = 8+− Excellent** ***M* = 6–7.99 - Good** ***M ≤6* - Poor**	*M=* 6.68; *SD=* 2.10

#### 2.4.2 Secondary outcomes

Secondary outcomes included physical, psychological, social, and environmental QoL, depression, anxiety, pain intensity, and pain interference. We assessed QoL domains with the WHOQOL-BREF [21], with higher scores on domains representing higher QoL (domain total score range = 4–20). We assessed depressive symptoms with the Patient Health Questionnaire for Depression (PHQ-9; total score range = 0–27) [22], and we assessed anxiety with the Generalized Anxiety Scale (GAD-7; total score range = 0–21) [23]. Higher scores on these measures indicate higher emotional distress. We assessed pain intensity with the Graded Chronic Pain Scale, with higher scores indicating greater pain experience (total score range = 0–60) [24]. We assessed pain interference with the PROMIS Pain Interference-8a scale, with higher scores indicating greater pain interference with functioning (total score range = 8–40) [25]. All measures have been extensively validated in medical populations with specific details of each measure found in the study protocol paper [26] and the original 3RP-NF publication [11]. Measures were selected to allow for comparisons between NF-Web outcomes to other NF-Web trials [11].

### 2.5 Statistical methods

We used the Statistical Packages for the Social Sciences version 25 software (SPSS 25) to analyze quantitative data similarly to the 3RP-NF parent trial [11,[26], [27]]. As elaborated on in Table 3, we assessed feasibility (*N*/%) by examining ratios of enrollment, measurement, and treatment adherence. We assessed acceptability (*M/*SD) by examining total scores of participants who completed the NF-Web program and provided posttest data. We explored each secondary outcome in NF-Web from baseline to posttest and measured each descriptively (*N, %, M, SD*) as well as inferentially (paired-samples *t-*tests) for signals of improvements. Given the limited sample size of this open pilot study, we were not sufficiently powered to identify any differences in quantitative outcomes between cohorts or across demographic variables.

## 3 Results

### 3.1 Participant characteristics

Across the entirety of the study period, we received 69 inquires for participation (34 for cohort 1 and 35 for cohort 2). Fifty-seven of those inquiring met telephonically with the study coordinator for a screening call and 35 of those prospective participants screened met eligibility criteria. Twenty-eight of those eligible consented and enrolled in the study (14 per cohort; *N* = 28). Our final sample included 19 people with NF1 (67.86%), 6 people with NF2 (21.43%), and 3 people with SCHW (10.71%). The sample was majority female (78.6%) and White (82.1%). Half of the sample (*n* = 14*;* 50.00%) was married, followed by those who reported being single (*n* = 10; 35.7%). Learning disabilities were common in the sample, with 8 participants (*n* = 8; 28.6%) reporting confirmed diagnoses and 4 (*n* = 4; 14.3%) reporting suspected disability without a formal diagnosis. Participant demographics are presented in Table 4.

**Table 4:** Demographic characteristics for NF-Web sample (*N* = 28).

Characteristics		N (%)
Diagnosis		
NF1		19 (67.86%)
NF2		6 (21.43%)
Schwannomatosis		3 (10.71%)
Age		*M* = 45.86 (*SD* = 13.67)
Gender		
Female		22 (78.6%)
Male		6 (21.5%)
Marital Status		
Married		14 (50.00%)
Living With Someone		1 (3.57%)
in a Committed Relationship		
Single		10 (35.71%)
Separated		1 (3.57%)
Divorced		1 (3.57%)
Widowed		1 (3.57%)
Education Level		
Completed high school		5 (17.9%)
Some college or associate degree		12 (42.9%)
Completed 4 years of college		6 (21.4%)
Graduated of Professional Degree		5 (17.9%)
Learning diagnosis		
Yes, I Was Diagnosed with One		8 (28.57%)
I Think So, I Was Never Formally Diagnosed		4 (14.29%)
No		14 (50.00%)
I Don't Know		2 (7.14%)
Race		
White		23 (82.14%)
Black/African American		1 (3.57%)
More Than One Race		2 (7.14%)
Missing/ choose not to answer		2 (7.14%)
Ethnicity		
Hispanic or Latino/Latina		2 (7.14%)
Not Hispanic or Latino/Latina		25 (89.29%)
Missing/ choose not to answer		1 (3.57%)

### 3.2 Feasibility markers

A priori benchmarks for feasibility and acceptability were met for the NF-Web trial. Twenty-eight participants (*N* = 28; 100% of sample) of the 35 eligible enrolled in the study (80%; enrollment). All 28 participants completed baseline assessments and completed in at least one module of NF-Web (100%) and 25 completed posttests (89.3%; measurement) and 22 participants completed exit interviews (78.6%). Twenty participants completed at least half (≥4) of the modules (71%; treatment adherence), the average number of modules completed during the 8 weeks was 5.1 (SD = 2.7), and as a trend, participation decreased toward the last 2–3 weeks of the program. Video lessons and homework completion were Fair to Good, with 58.0% of assigned video lessons being watched and 70.9% of assigned homework tasks being completed throughout the course of the 8-week program (treatment adherence). Participant satisfaction (*M* = 8.85 of 10; SD = 2.35), credibility (*M* = 7.07 of 10; SD = 1.44), and expectancy (*M* = 6.68 of 10; SD = 2.10) were all considered Good to Excellent for web-based trials (acceptability). Definitions, benchmarks, and qualifiers, as well as NF-Web outcomes can be examined in Table 3.

### 3.3 Secondary outcomes

NF-Web participation was associated with positive changes in all domains of QoL, including physical (*M diff* = +1.74, *p* = 0.005, *d* = 0.73), psychological (*M diff* = +2.06, *p* < 0.001, *d* = 0.67), social (*M diff* = +1.60, *p* = 0.007, *d* = 0.49), and environmental (*M diff* = +1.20, *p* = 0.012, *d* = 0.55) from pre-to posttest. NF-Web participation was associated with positive changes in emotional distress, including depression (*M diff* = −2.57, p = 0.012, *d* = 0.43), anxiety (*M diff* = −3.28, *p* < 0.001., *d* = 0.69), and stress (*M diff* = −3.04, *p* = 0.018, *d* = 0.41) from pre-to posttest. There were no significant differences from pre-to-posttest for pain intensity (*M diff* = −1.33, *p* = 0.700, *d* = 0.05) or pain interference (*M diff* = −1.82, *p* = 0.325, *d* = 0.18). Table 5 displays secondary outcomes.

**Table 5:** Unadjusted baseline and posttest outcomes (M/SD and Cohen's *d*) after NF-Web Participation.

Measurement	Baseline (T1)	Posttest (T2)	*M Diff (T2-T1)*	Effect Size (Cohen's *d*)
**Physical Health QoL**	14.20 (1.78)	15.95 (2.90)	1.74	0.73
**Psychological QoL**	12.25 (2.97)	14.31 (3.13)	2.06	0.67
**Social relations QoL**	13.24 (3.25)	14.84 (3.30)	1.60	0.49
**Environmental QoL**	15.61 (1.91)	16.80 (2.40)	1.20	0.55
**Depression**	10.00 (5.62)	7.43 (6.21)	−2.57	0.43
**Anxiety**	8.40 (5.13)	5.12 (4.40)	−3.28	0.69
**Stress**	19.46 (6.78)	16.42 (7.98)	−3.04	0.41
**Pain intensity**	40.67 (28.56)	39.33 (28.85)	−1.33	0.05
**Pain Interference**	19.59 (10.00)	17.77 (10.16)	−1.82	0.18

## 4 Discussion and conclusion

### 4.1 Discussion

This is the first study to examine early feasibility, acceptability, and signals of improvement of an individually delivered asynchronous web-based treatment based on the 3RP-NF for adults with NF. NF-Web met all a priori feasibility (i.e., enrollment, measurement, and treatment adherence) benchmarks (Fair to Excellent [>50%]) similar to our prior small sample psychosocial trials [10,19,20,28]. NF-Web acceptability outcomes (i.e., credibility, expectancy, and satisfaction) also met a priori benchmarks (Good to Excellent; [*M* > 6]). Taken together, these initial findings are promising – specifically that NF-Web is a feasible and acceptable program for adults with NF and could be a potential treatment option in the future, especially for participants who cannot or prefer not to participate in the live video trial.

Within-group improvements in secondary outcomes, including QoL and emotional distress, were consistent with our previous feasibility trials in NF and suggest that NF-Web demonstrates signals of improvement for psychosocial outcomes [[10], [11],^29^]. These outcomes of QoL and emotional distress are two impactful variables to address for participants in a psychosocial intervention and may even produce downstream benefits for participants in other areas of functioning (e.g., NF-specific stressors, social isolation) [11,30]. Pain did not improve after participating in the NF-Web program. This finding may be partially due to the NF-Web not being a pain-specific intervention, and pain also typically presenting heterogeneously in this population [31,32]. With larger samples, pain outcomes can be examined by NF types – understanding which patients are most positively impacted by the program skills. Future fully powered trials may also examine changes in treatment targets (e.g., mindfulness, coping, social support) in addition to QoL and emotional distress, and how these changes in treatment targets fare against the live video, group 3RP-NF program. With more accessible options for psychosocial care, adults with NF can pursue treatments which aim to improve other aspects of their overall health beyond the biomedical burdens of the illness. We believe that NF-Web provides a positive step in this direction.

Our study limitations included a small sample size, no comparison group, a mostly homogeneous sample (white, female, well-educated), and a lack of power to compare findings by demographics (e.g., gender, NF type). These limitations are similar to other pilot studies [33], including several of our own previous studies in NF [10,19]. Future studies may benefit from partnering directly with the NF community (clinics, organizations, foundations) in creative ways (e.g., word-of-mouth and snowball recruiting, “NF-Web mentors” from diverse backgrounds) to reach more diverse participants.

Additionally, given the similarity of NF-Web to online courses with videos, homework assignments, and quizzes, a possible limitation may be that participants who do not prefer this medium for learning and skill acquisition may have been detoured from participating. Anecdotal accounts from this sample (exit interviews) suggest that those choosing to participate in NF-Web did not experience these aspects of the program as deterrent, however when considering this program for a wider audience other program methods may be explored. Relatedly, our sample had a relatively high number of patients with learning disabilities which may impact the generalizability of these findings.

Despite using multiple modalities (e.g., audio/video, written materials), future studies may benefit from inquiring how one's learning disabilities and/or differences impact their participation in technology-based interventions. Also, the GCPS was used for assessing pain intensity, which deviates from the Response Evaluation in Neurofibromatosis and Schwannomatosis (REiNS)-recommended measures. Last, we did not systematically measure engagement in the NF-Web program during the 8 weeks as part of this study. With the social support aspect being the most notable difference between the two programs (live video and web-based), future studies will attempt to examine how key aspects of the NF-Web program (e.g., discussion board, online participant interaction) can facilitate engagement and how other methods of participation (e.g., linking participants to online and local NF communities) can be encouraged. Despite these limitations, our study strengths included early feasibility testing a novel web-based program for an underserved population which appeared to increase enrollment, participation, accessibility, as well as retention throughout the study course.

### 4.2 Innovation

Web-based programs are valuable for individuals who prefer to learn skills on their own timeline, have barriers to live video participation, and who may also have apprehensions about interacting with others during treatment. Although these do not impact other important reasons for limited participation (e.g., forgetfulness, low motivation), offering multiple program types in various modalities, we can increase accessibility to psychosocial care and make these kinds of programs sustainable in a time of limited mental health resources across the world. Long-term, multiple forms of the same evidence-based treatment can complement one another and can even supplement one another if needed (e.g., completing an NF-Web module if a participant misses a live video session; “step-down” treatment options based on participant treatment needs/desire).

### 4.3 Conclusion

Overall, NF-Web demonstrates early feasibility and acceptability for participants who are looking for a point of entry into these psychosocial interventions who may not be ready to commit to the live video or in-person group options. Having a web-version of this program provides more support to this underserved and in-need population of adults with rare illnesses. Clinicians can work with patients to decide which treatment modality fits their needs best and address barriers they may experiences when seeking psychosocial care. The next phases of this study will work to address the above limitations and plan to include a comparison group to examine between group effects for NF-Web.

## Conflicts of interest

All authors declare that they have no conflicts of interest or competing interests related to this manuscript.

## Funding

Dr. Ethan Lester was directly supported by foundation grants-in-aid (early career) from NF Northeast, NF Midwest, Texas Neurofibromatosis Foundation, and the 10.13039/100001545Children's Tumor Foundation.

## Ethical approval

All procedures performed in studies involving human participants were in accordance with the ethical standards of the institutional and/or national research committee and with the 1964 Helsinki declaration and its later amendments or comparable ethical standards. This article does not contain any studies with animals performed by any of the authors.

## Informed consent

Informed consent was obtained from all individual participants included in the study.

## Credit author statement

Description of authors' contribution to the work:

Lester: Conceptualization, Data curation, Formal analysis, Funding aquisition, Investigation, Methodology, Project administration, Resources, Supervision, Writing - original draft, Writing - review and editing.

Fishbein: Project administration, Writing - original draft, Writing - review and editing.

Peterson: Writing - original draft, Writing - review and editing.

Vranceanu: Writing - original draft, Writing - review and editing, Methodology, Supervision.
